# Good Places to Live and Sleep Well: A Literature Review about the Role of Architecture in Determining Non-Visual Effects of Light

**DOI:** 10.3390/ijerph18031002

**Published:** 2021-01-23

**Authors:** Laura Bellia, Francesca Fragliasso

**Affiliations:** Department of industrial Engineering, University of Naples Federico II, Piazzale Tecchio, 80, 80125 Naples, Italy; francesca.fragliasso@unina.it

**Keywords:** architecture and lighting, circadian lighting, daylight, integrative lighting, literature review, non-visual effects of light

## Abstract

Light plays a crucial role in affecting the melatonin secretion process, and consequently the sleep–wake cycle. Research has demonstrated that the main characteristics of lighting affecting the so-called circadian rhythms are spectrum, light levels, spatial pattern and temporal pattern (i.e., duration of exposure, timing and previous exposure history). Considering that today people spend most of their time in indoor environments, the light dose they receive strictly depends on the characteristics of the spaces where they live: location and orientation of the building, dimensions of the windows, presence of external obstructions, geometric characteristics of the space, optical properties of walls and furniture. Understanding the interaction mechanism between light and architecture is fundamental to design non-visually comfortable spaces. The goal of the paper is to deepen this complex issue. It is divided into two parts: a brief historical excursus about the relationship between lighting practice and architecture throughout the centuries and a review of the available research works about the topic. The analysis demonstrates that despite the efforts of the research, numerous open questions still remain, and they are mostly due to the lack of a shared and clear method to evaluate the effects of lighting on circadian rhythm regulation.

## 1. Introduction

It is commonly accepted that light has multiple effects on people, not limited to visual perception, but consisting in human responses of different types: circadian responses (referring to the regulation of the biological processes based on a 24 h cycle), neuroendocrine responses (referring to hormone production) and neurobehavioral responses (referring to the relationship between the action of the nervous system and human behavior) [1]. Practically, this means that light influences aspects like body temperature regulation [2,3], heart rate modulation [4], variation in alertness state [5], mood [6], work performance [7,8] and, of course, melatonin suppression and, consequently, the sleep–wake cycle [9,10]. 

Therefore, a proper exposure to light is fundamental to synchronize circadian rhythms and guarantee sleep quality. For this reason, several studies tried to understand what light characteristics mostly affect the activation of circadian responses. Although there are many still-open issues, according to the current knowledge, the essential parameters to consider are: spectrum, light levels, spatial pattern and temporal pattern, the latter referring to three different aspects, i.e., duration of exposure, timing and previous exposure history [1]. The photoreceptors mainly responsible for non-visual effects of light, intrinsically photosensitive retinal ganglion cells (ipRGCs), are more sensitive to short wavelengths, being characterized by a spectral sensitivity curve peaking at 490 nm [11]. Therefore, light sources with a spectral power distribution (SPD) concentrated in this part of the spectrum are more effective in stimulating the circadian system. The intensity of the source is critical as well. For example, in the case of white light commonly used in working environments, illuminance at the eye level has much greater potential than the spectrum to affect non-visual response [1]. Previous studies attempted to determine the light dose (in terms of eye illuminance) necessary to induce an alert state and reduce sleepiness by means of nighttime [12] and daytime [13] experiments. However, it was not possible to give an absolute answer, since when non-visual effects are considered, temporal aspects are essential. Indeed, it has been demonstrated that the circadian system reacts differently to light stimuli, depending on the time of day, and that light can both phase advance the circadian clock to an earlier time or phase delay it to a later time [14]. This dependency on timing is defined by a phase response curve (PRC). Although this response is different among individuals, according to Khalsa et al. [15], phase delays occurred when the light stimulus was centered before the critical phase, when the core body temperature reached its daily minimum value; on the contrary, phase advances occurred when the light stimulus was centered after the critical phase, and no phase shift occurred at the critical phase. Considering an individual typically sleeping from 00:00 to 08:00, the minimum core body temperature time is around 06:00, and the day can be subdivided into different intervals: early to mid-morning (06:00–10:00 h), when sufficient illuminance can serve to phase advance the clock in the majority of people; mid-morning to early evening (10:00–18:00 h), when high illuminance levels may lead to increased subjective alertness without exerting substantial phase shifting effects; the last is notional nighttime (18:00–06:00 h), when light exposure that might trigger non-visual effects is to be avoided, so as not to disrupt the natural wake–sleep cycle [16]. Of course, this is not applicable to some categories of people, for example, shift workers. Moreover, it has been demonstrated that the circadian response is different depending on the duration of light exposure [17] and that it is affected by the previous photic history as well: a light stimulus in a specific moment of the day has diverse effects on people depending on the light dose they have previously received [18,19]. The spatial pattern of light, i.e., the luminance distribution in the space is important as well, since it determines the corresponding light pattern on the retina. It has been proved that the inferior retina contributes to the light-induced suppression of melatonin more than the superior retina [20] and that there is a significant difference in sensitivity between exposure of the lateral and nasal parts of the retina, showing that melatonin suppression is maximal upon exposure of the nasal part [21]. Of course, the retinal light pattern can significantly change when people move the gaze from one direction to another [22].

Another aspect deserving to be investigated is the identification of the factors ensuring that people receive the “proper dose of light” (in terms of quantity, quality and timing) during the day. Even though that is a complex topic, it can be asserted that the received light dose depends on how many hours and in what moment of the day a person sleeps or is awake, and, when they are awake, on how much time they spend outside or inside buildings. That gives a measure of how much time they are exposed to daylight, electric light or both. 

In the pre-industrial age, people spent most of the day outside, busy in their activities which were primarily agricultural, constructive or craft based and were substantially limited by the sunrise and the sunset [22]. The invention of electric light, industrial development with the consequent urban densification, the evolution of construction techniques and the improvement of indoor climate control technologies have inverted this paradigm, moving most people’s activities inside buildings [22]. To have an idea of how much time people spend outside, we can refer to a study performed in Chicago that has estimated that office workers, leading a typical life, receive about 2.5 h of bright outdoor light (>1000 lux) on summer workdays, but only about half of this on winter workdays [23]. Undeniably, the “daily light diet” of contemporaneous people is different than that of their ancestors, being based on a lower dose of daylight and a significant amount of electric light. That implies a completely different stimulation of the circadian system, creating the risk that people are not exposed to very robust dark–light cycles [24,25]. Indeed, outdoor daylight is 5–10 times more intense than indoor light at a typical workplace, it has a spectrum completely different from those of the electric sources and it determines different patterns and varies in intensity and color temperature within the 24 hour cycle as well as between the various seasons of the year [26]. 

Based on all these considerations, it becomes clear that architecture, as the discipline with the aim of the construction and the organization of space, has an essential role in defining the non-visual comfort conditions of people living in buildings. The amount of daylight entering a space depends on the type, dimensions and orientation of the apertures, and on the presence of external obstructions. The conformation of the luminous environment varies, according to the geometric characteristics of the space and on optical properties of the surfaces; the spectral composition of the light reaching observers’ eyes is due not only to the nature of the source, but also to the optical characteristics of walls and furniture. In other words, the architectural space shapes light, modifying all those characteristics (intensity, spectrum, directionality) fundamental for the entrainment of the circadian system. This can be expressed by saying that each space possesses a specific “circadian potential” [16], i.e., the capability to define a luminous environment more or less comfortable from the non-visual point of view. This capability is so significant that Andersen et al. in [27] wrote: “*Architecture and the eye perform parallel roles in regulating light input to the brain. Just as the pupil fine-tunes the amount of light that enters the eye, the intensity and quality of light reaching internal surfaces are determined by the size and shape of building openings, the light-transmitting qualities of the glazing chosen, and the presence and sizing of shading*”.

Understanding the interaction mechanism between light and architecture is fundamental to design non-visually comfortable spaces, and strategies to increase circadian light exposure should be considered in architectural design [28]. Despite that, it is not easy to precisely estimate the role of architecture in contributing to the non-visual effects of light and to define the weight of each factor (orientation, dimension of windows, optical characteristics of surfaces, etc.) in altering daylight and electric light characteristics. 

The goal of the paper is to deepen this complex issue. For this purpose, the investigation is divided into two parts: (1) a brief historical excursus about the relationship between lighting practice and architecture throughout the centuries; (2) a review of the available research works reporting experiments aiming at understanding the effects of architecture on lighting, considering the non-visual implications. The former part will be useful to underline how awareness about the beneficial effects of light has been changing throughout history and to understand how much the need to guarantee well-lit indoor spaces has influenced architecture; the latter will provide a detailed description of all the architectural features able to modify light characteristics fundamental for circadian entrainment, i.e., light levels, spectrum, temporal and spatial patterns. Moreover, it will be useful to highlight the aspects related to this research field that still need to be further investigated and deepened. 

## 2. Method

As previously mentioned, the paper is divided into two parts: a historical excursus and a review of the current research.

The complex topic of the relationship between architecture and lighting throughout the centuries deserves a deep and specific treatise. It can be considered by itself a specific field of research that couples expertise on both history of architecture and lighting. Despite that, it is considered useful to insert a brief excursus about this topic (not presuming to be exhaustive), trying to outline in broad terms the evolution of the link between architecture and lighting in three specific historical macro periods, according to the classification reported in [29]: pre-industrial age, industrial age and post-industrial age. The excursus is useful to underline the strong interconnection between lighting and architectural design and will be presented in Section 3.

The second part of the paper, i.e., the deep analysis of the current research about the role of architecture in defining the non-visual effects of lighting, will be reported in Section 4. For this part, the literature review has been performed by means of the Scopus search engine. The research was limited to engineering papers and the following keywords were used: circadian light, human centric lighting, integrative lighting, non-image-forming effects of light, non-visual effects of light, circadian stimulus, equivalent melanopic. The keywords were generic to expand as much as possible the research and, of course, they provided a large amount of papers. Specifically, the number of results for each keyword (duplicates included) were, respectively: 485, 56, 25, 24, 90, 90 and 7. From all these articles, only those specifically regarding architectural feature effects on the circadian system were selected. This means that the following were excluded: studies analyzing non-visual effects from a general point of view (for example, reviews or opinion articles); articles proposing calculation methods to evaluate non-visual effects; papers considering visual responses different from the effects on circadian rhythms; works describing exclusively how light source characteristics (emitted flux, Correlated Color Temperature -CCT-) influence non-visual responses, since they do not refer to the interaction of light with architectural elements. This selection process strongly limited the final amount of analyzed papers, demonstrating that the study of the interaction between architecture and light in the context of non-visual effects is still a fertile field of research to be explored.

Considering the complexity of the analyzed topic, for reasons of convenience in the exposition, Section 4 will be organized in the following way. First of all, based on what was reported in the collected papers, the architectural factors influencing circadian system regulation will be listed and classified according to the design scale (urban, architectural, technological and interior design). Moreover, for each scale, the involved lighting characteristics will be identified (light levels, spectrum, temporal and spatial patterns). Then, a more accurate description of the collected papers will be provided in two subsections: Section 4.1 is related to urban and architectural design and Section 4.2 is about technological and interior design. To make the comparison among the analyzed studies easier, most of the selected papers, representative of the different architectural factors, will be described in depth by means of recapitulatory tables. In these tables, for each paper, details about the research goal and the research method will be given. As for the goal, the following information will be provided: primary goal (sometimes analyzed works are not focused exclusively on the interaction between light and architecture) and the analyzed architectural parameters (e.g., window dimensions, orientation, optical characteristics of surfaces, etc.). As for the method, all the specific details about the research procedures will be provided: characteristics of the case-study, features of the light sources, method to evaluate the circadian effects of light, analysis typology (numerical simulations or field measurements). Moreover, the metrics adopted for quantifying circadian effects of light are reported: circadian action factor, circadian stimulus, equivalent melanopic illuminance. To help with the reading of the paper, an explanation about these parameters is reported in Appendix A.

## 3. Lighting and Architecture over Time

As mentioned in the Introduction, architectural design is fundamental in defining the lighting characteristics of indoor spaces. Till the invention of electric light in the second half of the nineteenth century, daylight was the primary source to light indoor environments. Even if daylighting, as a formal subject of architectural study, was born in northern Europe in the late nineteenth and early twentieth centuries [30], one could say that “*the history of architecture is synonymous with the history of the window and of daylighting*” [31]. According to Boubekri [30], it is possible to recognize an innate understanding of the importance of the presence of the sun in dwellings, testified by abundant examples in the history of architecture. However, as the author himself affirms, the relationship between daylighting principles and those of construction has not always been genuine throughout history. Only recent decades have architects and urban planners gained a renovated consciousness about the importance of daylighting principles. In accordance with this concept, Chepchumba in [29] identifies three periods that outline the history of daylighting: pre-industrial, industrial and post-industrial ages. Although this classification expressly refers to daylight, it can be considered fitting to describe more generally the relationship between lighting and architecture, as described in the following sections.

### 3.1. Pre-Industrial Age

During the pre-industrial age, daylight was the primary light source and the link between daylighting principles and architectural ones was so strong that the building shape was conceived as a natural response to the need to provide air, heat and daylight in interior spaces. This awareness about the benefits of the sun, shown thorough the way architects shaped dwellings and carved openings, was evident in different antique civilizations: in the ancient city of Macchu Picchu, known as the “lost city of the Incas”, the main façades of buildings primarily faced east and south to maximize solar exposure; from around AD 600 to AD 1200, ancient people populating the niches and alcoves of the canyons of Utah, Arizona and New Mexico built their houses as complexes of multistoried buildings assembled in terraces and opened towards the sky and sunlight for winter heating, while the upper edges of the towering cliffs provided shade during the hot summer [30]. This intimate relationship between architecture and nature, and therefore daylight, was typical of Greek and Roman culture as well. For example, typical houses in Greece were composed of two sections; a southern one, occupied mostly in winter, and a northern one, occupied mostly in summer. In this way, it was possible to optimize the indoor comfort conditions of the inhabitants. The attention to solar radiation harvesting was present in ancient Roman buildings as well, in which a strong correlation between the function of the rooms and window size existed. The representative spaces of the houses, used as dining rooms, living rooms or studios were the most daylit ones, whereas those destined for service activities were almost dark [32]. In the famous work “De Architectura” (liber sextus) by Vitruvius [33], it can be read that winter triclinia and baths must be oriented towards the west, in order to receive afternoon light; bedrooms and libraries must face east, in order to receive light early in the morning and for the control of humidity; during spring and fall, triclinia should face east, and during summer, north, to avoid glare and summer overheating. Moreover, painters’, weavers’ and embroiderers’ workshops should always receive daylight from the north, avoiding sunlight glare and obtaining uniform light patterns. Indeed, the big villas and domus, owned by very rich people, were provided with big atria and peristyles where several rooms faced the different orientations, allowing them to change the position of triclinia, and even bedrooms, according to the season. 

### 3.2. Industrial Age 

The connection between daylight and architecture was strong till the industrial revolution. With the development of the manufacturing production process, a massive amount of people left rural areas and moved to urban centers to seek work [30,31]. The demands for housing rapidly increased and the uncontrolled urbanization determined the spread of densely populated buildings facing narrow streets, where sanitary conditions were terrible and sunlight exposure was not guaranteed. The spread of common residential constructions, produced cheaply and lacking in comfortable condition, led to outbreaks of cholera, typhus, rickets, and tuberculosis. The medical analyses conducted in that period, aiming at identifying the reasons for the epidemics, recognized that the cause of the outbreaks was represented by the foul waters from open sewers, providing the chief environment for pathogens. However, other environmental elements could be recognized as exacerbating factors, among was which the lack of sunlight in buildings [30]. This terrible urbanistic and social crisis motivated the action of reformers and urban planners. The reformers underlined the necessity for governments to take the responsibility for the protection and safeguarding of public health and welfare, accounting for the strong connection between people’s wellness and the characteristics of the spaces they lived in [34]. In parallel, the effort of urban planners had been focusing on the proposal of new urban models for the construction of the so-called cities of health, populated by buildings surrounded by green spaces and characterized by healthy indoor conditions where sunlight access was a mandatory requirement [35,36]. This attention to the dialogue of architecture with the elements of nature persisted between the nineteenth and the twentieth centuries, arriving at the modern movement; its notable exponents, such as Frank Lloyd Wright, Le Corbusier, Louis Kahn and Alvar Aalto, retained many of the historical principles of site orientation, natural ventilation and daylight illumination, interpreted from a new point of view thanks to the introduction of new construction technologies [29]. Indeed, the spread of frame structures made the exterior walls free from the function of bearing loads, reducing them to closing elements. That made possible the easy realization of big windows, bringing air and daylight inside buildings and allowing views out. The use of ribbon windows, one of the *five points of architecture* theorized by Le Corbusier [37], can be considered a perfect example of this new way to conceive the relationship between daylight and architecture. In the meantime, another event occurred and assumed a crucial role in affecting daylighting practice during the long period indicated as the industrial age: the invention of electric lighting at the end of the nineteenth century. As was previously mentioned, for centuries, before the introduction of electric light, daylight was the primary source to light indoor environments; oil lamps and candles were used as supplementary illuminations. The introduction of electric light, not only completely changed people’s habits, as reported in the Introduction, but also had a strong role in changing the way to build edifices. The change of social structure and job organization determined the development of buildings for work activities, such as factories and offices, where people stayed for most of the day. These spaces were often characterized by open-plan rooms, which needed long and big windows in order to be properly daylit. However, the need to reduce the volume of edifices for economies of structure caused a reduction of the height of indoor spaces, negatively affecting indoor daylight distribution. Electric light seemed the best solution to solve these problems, especially considering that the utility companies and the manufacturers kept up the pressure to increase the consumption of electricity and the sale of lamps and equipment [30,31]. By the 1960s, the idea that electric light could become the primary source substituting daylight was spread. As can be read in [38]: “*It is inevitable that artificial light must become the primary light source where efficiency of vision is combined with an economic analysis of building function. Natural lighting is becoming a luxury*”. This led to windowless factories and even windowless schools [31]. 

### 3.3. Post-Industrial Age

The post-industrial age, with the energetic crisis following the oil embargo of 1973, demonstrated that a turnaround was necessary. It was recognized that the construction sector caused a lot of pressure on the world’s energy costs (36% of final energy use in 2018 according to [39]) and that it was necessary to apply effective strategies to reduce energy consumption related to building management. Of course, the use of renewable energy sources for heating, cooling and lighting buildings was one of these strategies. So, architects and engineers started to concentrate their efforts on finding ways to optimize the use of the so-called green energy sources, on one hand experimenting with new technologies, and, on the other hand, getting back the cultural inheritance hidden inside ancient buildings, clear proof of a safe relationship between nature and the built environment [40]. A study of vernacular architecture provided practical teachings about the optimization of the energy performance of buildings at a low cost, using local sources and materials [41]. It provided an ante-litteram demonstration of what is defined as bioclimatic architecture, i.e., a constructive culture based on passive solar technologies, i.e., heating or cooling techniques that passively absorb (or protect from) the sun’s energy and contain no moving parts [42]. In this context, daylighting practice has gained a new importance in building design: the use of daylight allows for reducing the hours of electric lighting and reducing related costs [43,44,45]. 

However, although the energetic issue has been the primary issue driving the renewed attention to daylighting use, its beneficial effects on visual and non-visual comfort have today gained importance as well, thanks to results from recent research, and the consciousness about the necessity to keep the balance between energy and wellness aspects which is now considered fundamental [46]. This is demonstrated by the effort of specialized associations in spreading awareness among lighting designers about the need to substitute the typical quantitative design approach with a qualitative one, accounting for all the visual and non-visual implications of light. This effort has culminated in the recent introduction by CIE of the concept of integrative lighting, defined as “*Lighting specifically integrating both visual and non-visual effects and producing physiological and/or psychological benefits upon humans*” [47,48].

## 4. Architectural Features Affecting Light Characteristics

The brief excursus reported in Section 3 has demonstrated that a wise configuration of the architectural space can optimize daylight distribution in indoor environments even without using complex technologies. Of course, it must not be forgotten that the configuration of the environment modifies not only the daylight characteristics, but also those of electric light: for example, just think about the variation in the luminous pattern obtained by changing the color of the walls. 

**Figure 1:**
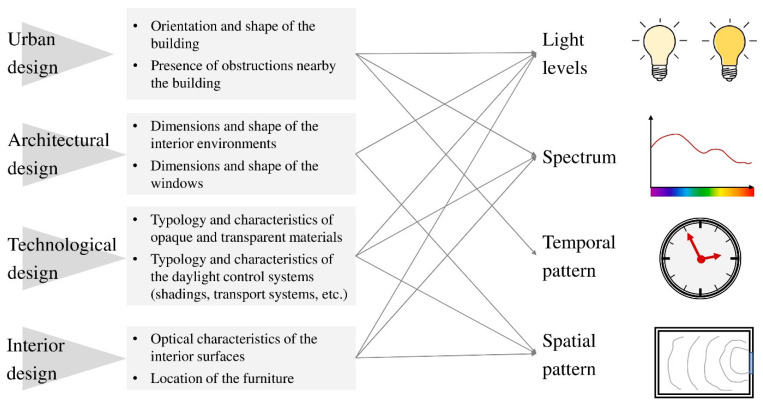
Design factors modifying light characteristics, in turn affecting circadian systems.

As will be deepened in the following subsections, research has underlined that there are several architectural factors affecting indoor lighting characteristics essential for circadian system entrainment (light levels, spectrum, temporal and spatial patterns). So, before analyzing the main research results about this topic, it seems useful to list all the factors that have been analyzed by researchers. They are schematized in Figure 1 and classified according to the design scale, to underline that the link between light and space must be managed at each stage of the design process. 

Urban design defines the location of buildings in the territory, their dimensions, shape and orientation, the mutual position and the location of green areas. As can be inferred from Figure 1, all these factors strongly affect daylight characteristics inside buildings, determining both the amount of light entering the spaces and the number of hours during which façades are irradiated [14]. For example, the relationship between the height of edifices and the distance among them is essential to guarantee proper daylighting on all the floors of the construction. Moreover, the edifice orientation is essential as well [49]: a building with the longitudinal axis oriented along the north–south direction is irradiated in a completely different way compared to the same building oriented according the east–west direction. Urban design also plays a significant role in defining the spectral composition of daylight entering the buildings. Indeed, daylight spectrum changes during the day [50,51] and, consequently, the spectral distribution of daylight irradiating east façades at sunrise will be different from that irradiating west façades at sunset. 

At the architectural scale, choices like the planimetric distribution of the spaces and the shape and dimensions of windows define both the amount of indoor daylight and its distribution [27]. The same space will be characterized by a different indoor luminous pattern if it is side-lit or if it is equipped with skylights [49]. Moreover, in an elongated rectangular room, the illuminance distribution will be more uniform if the windows are positioned on the long side and not on the short one. 

Technological choices about materials (both opaque [52] and transparent [53]) are essential as well. The configuration of the building envelope affects the amount of daylight (low visual transmittance values, presence of shading systems), the spectrum (use of non-neutral glass or of colored shading systems) and the indoor distribution (presence of redirecting systems like light shelves or of transporting systems like light tubes also lighting parts of the room far from the windows [54]). Other aspects to consider are the optical characteristics of façade materials [55]. Before entering indoor environments, daylight interacts with the outdoor space, changing its spectral characteristics depending on surfaces’ spectral reflectance; so, the daylight spectrum inside a building will be different if in front of it there is a line of trees or an edifice covered in bricks or in white plaster.

Finally, interior design choices have an impact on circadian effects as well since they modify characteristics of both daylight and electric light. This interaction not only will affect indoor light levels (a room with light walls will be more luminous than one with dark walls) [56], but also the spectrum, especially in spaces strongly characterized by colored (spectrally selective) surfaces [57]. Another aspect not to be neglected is the location of furniture. This is particularly relevant for spaces where people stay in the same position for a long time, such as health care facilities [58,59] or workplaces [60,61]. Workstation positioning is crucial in determining the amount and quality of light received by a worker from both luminaires and windows. A desk located next to the window will be more advantaged than one far away the perimetral wall, moreover, the luminance distribution in the visual field will be different if the workstation is oriented towards a window or towards a wall.

The following subsections analyze all these mentioned factors in depth, describing the results of current research. 

### 4.1. Urban and Architectural Design

Papers analyzing the interaction among light and urban/architectural design factors are described in Table 1.

**Table 1:** Papers analyzing factors related to urban and architectural design.

Ref.	Year	Goal: (GG) General Goal (IAF) Investigated Architectural Factors Affecting Circadian Response	Methodology			
			Description of the Case Study	Daylight Conditions: (L) Location (WF) Window Features	Evaluation of Circadian Effects: (ACP) Adopted Circadian Parameter (OP) Observer’s Position	Analysis Method: (TA) Type of Analysis (PA) Protocol of Analysis
[27]	2013	**-GG:** Proposal of a lighting simulation framework aiming to address how “circadian lighting potential” can become part of housing design or renovation processes. **-IAF:** Floor/window configuration, distance of measurement sensor from the window wall, wall reflectivity, type of blind usage, urban masking conditions and presence or absence of room partitioning.	The model of a typical historic Boston row house simulated according to a previous typological study. Thirty-two different trials were analyzed considering different spaces.	**-L:** Boston. **-WF:** The spaces were equipped alternatively with two or three windows, north- or south-oriented, diversely obstructed by other buildings.	**-ACP:** DA_circadian_ (circadian Daylight Autonomy). **-OP:** Eight vertical sensor planes (one per view direction) were modeled at a fixed eye-level height of 152.4 cm at two locations with different distances from the window.	**-TA:** Computer-based simulations. **-PA:** Dynamic daylight simulations were performed by means of Daysim (validated dynamic daylighting analysis software based on the radiance backward raytracer) to calculate vertical illuminances and then infer the DA_circadian_.
[49]	2014	**-GG:** Application of a new analysis methodology, for the evaluation of non-visual effects of daylight based on the use of dynamic daylight simulations, previously proposed in [16]. **-IAF:** Building location, window orientation and typology.	A residential dwelling where several spaces were analyzed: the living room, the kitchen, the entrance hall, small bathroom, large bathroom, the stairs to the basement.	**-L:** 8 European cities: Hamburg, Madrid, Paris, London, Rome, Warsaw, Moscow and Östersund. **-WF:** Each space was equipped with side windows of different shapes. However, two different configurations were analyzed: only side windows and side windows plus skylights. The building was oriented towards the four cardinal directions in turn.	**-ACP:** C_NVE_ (Cumulative Annual Non-visual Effect) evaluated at the observer’s eye level. **-OP:** The calculation points were located at head height in the analyzed spaces with a different density according to their dimensions. For each point, four different directions of view were considered.	**-TA:** Computer-based simulations. **-PA:** Dynamic daylight simulations were performed by means of radiance to calculate vertical illuminances at the calculation points, then the photopic illuminances were converted into N_VE_ (Non-visual Effect) values according to the methodology described in [16].
[60]	2014	**-GG:** Investigation of daylight characteristics, also considering the circadian impact of daylight entering three offices, to develop guidelines to better assess daylight quality in built environments. -IAF: Window dimensions and orientations, outdoor obstructions and indoor environment optical and geometrical characteristics.	Three real offices characterized by different dimensions, furniture and orientations: Office 1 (OF1)—3.89∙3.79∙3.00 m, Office 2 (OF2)—3.36∙ 4.35∙3.00 m, Office 3 (OF3)—2.79∙3.48∙2.8 m.	**-L:** Naples. **-WF:** OF1: French west-oriented window, dimensions 2.6∙2.1 m, shaded by a balcony overhang around 1 m; OF2: French east-oriented window, dimensions 2.6∙1.52 m, shaded by balcony overhang around 1 m; OF3: French south-oriented window, dimensions 2.44*1.53 m (no overhang).	**-ACP:** CS (Circadian Stimulus) at the observer’s position. **-OP:** A man sitting at the desk (1.2 m from the floor) looking forwards and 45° left and right. For each one of these directions, measurements were repeated for different tilt angles (0°, 15° and 15° downwards) to simulate different positions of the observer’s head.	**-TA:** Spectral measurements. **-TP:** Spectral irradiance was measured on several days from 31 May 2013 to 3 July 2013, with 1 h time steps from 10:00 to 18:00.
[61]	2014	**-GG:** Extending and enriching the findings of [60], comparing the previously performed summer measurements with winter ones. -IAF: As in [60].	As in [60].	**-L:** As in [60]. **-WF:** As in [60].	**-ACP:** As in [60]. **-OP:** As in [60].	**-TA:** Spectral measurements. **-PA:** Measurements were carried out on several days between late November 2013 and early February 2014, with 1 h time steps from 10:00 to 18:00.
[62]	2017	**-GG:** To determine percentage of days during which patients in typical hospital rooms would receive a minimum level of circadian stimulation. **-IAF:** Window-to-wall ratio, surface reflectance and latitudes.	A virtual room simulating a typical hospital room measuring 3.0∙6.0∙3.0 m. The model was simulated according to two reflectance schemes: a bright one characterized by 0.8, 0.6 and 0.8 reflectance values for walls, ceiling and floor, respectively, and a dark one characterized by 0.4, 0.2 and 0.6 reflectance values for walls, ceiling and floor, respectively.	**-L:** London and Madrid. **-WF:** Window was located in the short wall of the room. It is north oriented, and it was equipped with a double pane glazing characterized by 0.75 visual transmittance.	**-ACP:** Circadian stimulus autonomy evaluated according to a CS value equal to 0.35 considering as daylight spectrum the D65 illuminant. **-OP:** Two calculation grids were considered: a horizontal one to account for a lying patient and a vertical one to consider a sitting patient.	**-TA:** Dynamic daylight simulations. **-PA:** Dynamic daylight simulations were performed by means of Daysim considering the time range 8:00–12:00.
[63]	2019	**-GG:** To determine the suitable window size for multipurpose classrooms of educational buildings, in order to promote a proper CS value for students. **-IAF:** Window-to-wall ratio, surface reflectance, furniture reflectance.	A virtual room simulating a typical classroom measuring 3.0∙8.0∙8.0 m. The model was simulated according to two reflectance schemes: a bright one characterized by 0.8, 0.6 and 0.8 reflectance values for walls, ceiling and floor, respectively, and a dark one characterized by 0.4, 0.2 and 0.6 reflectance values for walls, ceiling and floor, respectively. The desk of the classroom was characterized in turn by three different finishes (white, light wood and light blue) modeled with different spectral reflectance values.	**-L:** London, Paris and Madrid. **-WF:** The room had a single 7.0 m long window of variable height located on one façade. The window had two different locations: centered in the façade or in an upper position with a height of sill of 1.5 m. It was equipped with a double pane glazing characterized by 0.75 visual transmittance. It was alternatively oriented towards north and south.	**-ACP:** Circadian stimulus autonomy evaluated according to a CS value equal to 0.1, 0.3 and 0.5. A different daylight spectrum was considered for each city based on statistical data on the percentage of overcast skies throughout the year. The spectrum at the eye level was the product of this spectrum to that of the modeled desks. **-OP:** A calculation grid composed of vertical sensors modeling the position of sitting students was used.	**-TA:** Dynamic daylight simulations. **-PA:** Dynamic daylight simulations were performed by means of Daysim.

When dealing with urban and architectural design, the connected lighting implications specifically refer to daylight. Indeed, Derek Phillips in [31] asserts that it is possible to outline a simple daylighting design based on: (1) decision on the siting of the building, taking into account orientation, sun path and the location of existing buildings or the landscape; (2) determination of room dimensions and subdivisions, (focusing especially on the relationship between height and depth, crucial for daylight penetration) based on the building function, considering present and future needs; (3) disposition and sizing of windows to guarantee the provision of view control of heat gain and glare; (4) installation of control systems, considering both those controls acting on the daylight entrance (regulating sunlight entrance to avoid glare and overheating) and those managing the electric lighting systems, guaranteeing the efficiency of the interaction between the natural and the artificial source. As can been seen, only the fourth point refers to the technological scale, underlining the strong connection between daylighting and urban/architectural design. 

Considering a non-visual point of view, this topic has been investigated by researchers with different methodological approaches. A group of works is specifically focused on the use of computer-based simulations [27,49,62,63] and aims at defining the combined effect of different factors on the circadian potential of indoor spaces: building location, floor/window ratio, window orientation and typology, type of blind, urban masking conditions, presence or absence of room partitioning. Moreover, the studies often evaluate the combined effects of urban/architectural parameters with interior design ones, such as the reflectance of the interior surfaces [27,62] and of the furniture [63]. In this last paper, analyzing the circadian potential of classrooms, the variation of light spectral distribution at the eye level due to the color of desks is considered. To do that, the desktop is simulated in three different finishes: white, light wood and light blue. One of the key topics of these studies is the proposal of a methodology to evaluate the effect in terms of circadian rhythm regulation, accounting for the extreme variability of daylight. Daylight availability is indeed assessed by means of a dynamic approach thanks to the use of software performing dynamic daylight simulations. Such software, starting from meteorological data contained in weather data files (weather archives collecting hourly data for an entire year and at a specific location about solar radiation, temperature, humidity, wind speed, etc.), models the sky luminance distribution according to the Perez sky model and, finally, accounting for the geometrical and optical characteristics of architectural surfaces, calculates indoor daylight illuminances with an hourly or sub-hourly time step for the entire year [64]. However, this modeling approach does not allow for obtaining information about daylight spectral composition, so circadian effects corresponding to a specific illuminance level are inferred using a standard CIE daylight illuminant. The circadian effects are then quantified by means of several parameters calculated for different positions and directions of view: circadian daylight autonomy (DA_circadian_) referring to specific daylight illuminance thresholds, in turn corresponding to acceptable “circadian” illuminances in [27], cumulative annual non-visual effect (C_NVE_) in [49], circadian stimulus (CS) and circadian stimulus autonomy (CSA) in [62,63] (for the parameters’ definitions, see the Appendix A).

Considering the amount of parameters contemporarily analyzed in each one of these papers, it is difficult to infer the weight each architectural factor has in affecting circadian rhythm regulation. However, the obtained results are useful to infer general considerations. For example, in [49], the large amount of collected data underlines that despite the several analyzed configurations, the C_NVE_ mostly depends on the orientation and typology of the window and on the direction of view. With side-lit windows, the privileged directions of view are those towards the windows, however, the introduction of skylights makes the results more homogeneous for all the directions of view. The orientation of windows affects not only the order of magnitude of the C_NVE_ values, but also the corresponding daily trend. Irrespective of the analyzed location, the orientation optimizing the C_NVE_ in the range 6:00–10:00 (the most useful for circadian entrainment) is east and that optimizing the C_NVE_ in the range 10-00–18:00 is south. These results underline the strong connections between urban/architectural design, defining the quantity of light entering indoor spaces, with the interior design, establishing the location of furniture, and, consequently, the typical positions of people in the space and the privileged directions of view. This connection between the different design scales is confirmed by [27], concluding that given a specific space, the variables most affecting the circadian stimulation were distance from the window and wall paint color.

Comparing the results obtained in these papers, it can be inferred that they are strictly connected to the specific location. On one hand, that makes it difficult to generalize the outcomes and, on the other hand, this aspect is a further demonstration of the necessity of wise daylighting criteria for building design. For example, Acosta et al., in [62], evaluating the CSA in hospital rooms located in two European cities, underline the differences in the building design criteria to apply to the two locations. In London, to guarantee a proper CS entrainment for 75% of the year for a patient lying in bed, considering a mean room surface reflectance close to 0.55, a window-to-façade ratio of 30% is necessary if the bed is close to the window, and of at least 60% if it is in the center of the room. In Madrid, a window-to-façade ratio of 40% is enough to meet the proposed CS criterion for 90% of the year in the middle zone of the room. The study analyzing classrooms located in Paris, London and Madrid [63] demonstrates that, regardless of the window size, orientation or location, the presence of low reflectance surfaces strictly reduces the CS. A white work plane allows an increase in CS of up to 30% compared to a pale blue desk for a large window—a 60% ratio—and up to 50% in the case of a small opening—a 30% ratio. In the studied locations with a white work plane, a window-to-façade ratio of 45% should be selected for the case of London and a minimum ratio of 30% is needed for Paris and Madrid. In accordance with the previous statement, the authors conclude that the environment reflectance is one of the most influential variables, just as the typical weather conditions are more decisive than latitude in promoting a good CS.

The effect of urban and architectural factors has also been studied with an experimental approach based on field measurements in real environments. Bellia et al. [60] evaluated the impact of window dimensions and orientations, outdoor obstructions and indoor environment optical and geometrical characteristics on circadian stimulus in three real offices located in Naples characterized by different dimensions, furniture and orientations. They performed spectral irradiance measurements at eye level with a one-hour time step during the typical working day in summer and then repeated the experiment in winter [61]. They found that despite the architectural differences of the studied rooms, eye-level spectral irradiances were similar in the three analyzed offices and did not vary much with the different sky conditions. Therefore, the registered variations of CS values can be mostly attributed to the different eye-level illuminances. Moreover, the comparison between eye-level spectral irradiances and D50, D55, D65 and D75 illuminants shows that eye-level irradiance trends tend to be similar in part to D50 trends and in part to D55 ones, while CS values tend to be closer to those calculated by using the D50 for the majority of the cases. The second experiment [61] confirms what was reported in the prior study. Moreover, it underlines that around 16:00 eye-level spectral irradiances are similar to D65 and D75 SPDs. This is probably attributable to the incoming sunset at that hour in winter. However, this result seems not to be particularly relevant for circadian stimulus calculation since, at that hour, illuminances are very low and electric light integration is required.

### 4.2. Technological and Interior Design 

In the case of papers dealing with technological and interior design, the effects of single factors on circadian rhythm regulation are easily assessed, since these works are, in general, focused on one specific aspect at a time. These factors modify not only daylight characteristics, but also electric light ones. For this reason, the works analyzed in this section are classified into two different tables: Table 2 reporting papers dealing with daylight and Table 3 referring to studies regarding electric light. 

**Table 2:** Papers analyzing factors related to technological and interior design focusing on daylight.

Ref.	Year	Goal: (GG) General Goal (IAF) Investigated Architectural Factors Affecting Circadian Response	Methodology			
			Description of the Case Study	Daylight Conditions: (L) Location (WF) Window Features	Evaluation of Circadian Effects: (ACP) Adopted Circadian Parameter (OP) Observer’s Position	Analysis Method: (TA) Type of Analysis (PA) Protocol of Analysis
[65]	2014	**-GG:** Evaluation of the color properties of internal perimetral walls and of external obstructions on circadian response, measuring daylight in scale models with surfaces characterized by different colors. **-IAF:** Spectral reflectance of indoor finishes; spectral reflectance of outdoor obstructions.	Four 1:5 scale models of office rooms, real dimensions equal to 3∙3∙10 m. In each box, the floor was dark brown, the ceiling white and the perimetral walls were painted in different colors: white (the reference), yellow, light gray, blue. The experiment was repeated twice: firstly, without obstructions and, secondly, posing an obstacle in front of the window colored light brown, providing a shading angle of 30 degrees.	**-L:** Bratislava. **-WF:** South-east oriented window, located on the short side of the room, equipped with a single clear glazing, real dimensions 2∙1.5 m.	**-ACP:** CL_A_ (Circadian Light) and CS evaluated at the observer’s eye level. **-OP:** A man sitting at 1/3 and 2/3 distance from the window, on the central longitudinal axis of the room, looking alternatively towards the window and to the south-west wall.	**-TA:** Field measurements. **-PA:** Measurements were performed on 31st of July 2014 starting from 2:30 p.m., immediately one after the other on a day characterized by a slightly cloudy sky.
[55]	2014	**-GG:** Evaluation of the color properties of external obstructions on circadian response, measuring daylight in scale models. **-IAF:** Spectral reflectance of outdoor obstructions.	Two 1:5 scale models of office rooms, real dimensions: 3∙3∙9 m. Indoor surfaces were all white. In front of the window an obstacle simulating a building wall was located, providing a shading angle of 30 degrees. The obstruction was colored in one case in dark red and in the other one in white.	**-L:** Bratislava. **-WF:** South-east-oriented window, located on the short side of the room, equipped with a single clear glazing, real dimensions 2∙1.5 m.	**-ACP:** CL_A_ and CS evaluated at the observer’s eye level. **-OP:** A man sitting at 3.3 m from the window and looking at it.	**-TA:** Field measurements. **-PA:** Continuous measurements were performed from 1st to 25th September 2013.
[66]	2014	**-GG:** Evaluation of the color properties of internal perimetral walls on circadian response, measuring daylight in scale models with internal surfaces characterized by different colors. **-IAF:** Spectral reflectance of indoor finishes.	Four 1:5 scale models of office rooms, real dimensions 3∙3∙9 m. In each box, the floor was dark brown, the ceiling white and the perimetral walls had different colors: white (the reference case), yellow, orange and red.	**-L:** Bratislava. **-WF:** South-East-oriented window, located on the short side of the room, equipped with a single clear glazing, real dimensions 2∙1.5 m.	**-ACP:** CL_A_ and CS evaluated at the observer’s eye level. **-OP:** A man sitting 3 m from the window, on the central longitudinal axis of the room, looking towards the window.	**-TA:** Field measurements. **-PA:** Measurements were performed on 26/3/2014 starting from 1:53 p.m., on a day characterized by a partly cloudy sky.
[67]	2016	**-GG:** Evaluation of the color properties of internal perimetral walls on circadian response, measuring daylight in scale models with internal surfaces characterized by different colors. **-IAF:** Spectral reflectance of indoor finishes.	Four 1:5 scale models of office rooms, real dimensions 7.5∙3∙3 m. One of the models was used as reference and the internal surfaces were completely white (floor, ceiling and walls), the other three had white ceiling and light brown floor, whereas the walls were different for each model: light gray, light blue and yellow.	**-L:** Bratislava. **-WF:** South-east-oriented window, located on the short side of the room, equipped with a single clear glazing, real dimensions 2∙1.5 m.	**-ACP:** CL_A_ and CS evaluated at the observer’s eye level. **-OP:** A man sitting at a distance from the window equal to 2.50, 3.75, 5.00, 6.25 m on the central longitudinal axis of the room, looking alternatively towards the window and to the south-west wall.	**-TA:** Field measurements. **-PA:** The measurements were performed on 15 January 2016 from 10:00 a.m. to 10:15 a.m. The sky was overcast without direct sunlight.
[68]	2019	**-GG:** To evaluate the effects of glazing type (color and transmittance) on participants’ alertness and mood, working performance and self-reported satisfaction in a full-scale office. **-IAF:** Spectral transmittance of glazing.	A full-scale office room, dimensions 6.3∙3.2∙3.8 m, matte neutral surfaces characterized by the following reflectance values: 0.3 (floor), 0.88 (wall) and 0.88 (ceiling).	**-L:** Beijing. **-WF:** One side window facing south, 2.3∙2.3 m, equipped alternatively with seven types of glazing: clear, blue, bronze, gray, green, dark blue and red.	**-ACP:** CL_A_ and CS at the observers’ eye level. **-OP:** Four men sitting at the same desk, two in front of the others with the window at their side.	**-TA:** Spectral measurements. **-PA:** Measurements were performed from 17 November 2017 to 15 January 2018 for 10 min each, from 8:30 to 16:00 with a 1.5 h lunch break in between. The measured positions were at the vertical plane near the participant’s eyes with an approximate height of 35 ± 5 cm above the table.
[69]	2020	**-GG:** To evaluate the impact of different glazing types and internal wall colors on the non-visual potential of daylight. **-IAF:** Spectral reflectance of walls and spectral transmittance of the glazing.	Typical cellular office, dimensions 3.0∙4.0∙2.6 m, reproduced as a 1:5 scale model and then as a virtual full-scale model. All internal surfaces except for the floor were white. The indoor wall colors were alternatively purple, blue, green, red, orange and gray at three different reflectance levels. Spectral characteristics of the scale model were measured and then imported into the simulation software.	**-L:** Ljubljana. **-WF:** Single north-oriented window, dimensions 1.4∙0.9 m. The window was equipped in turn with seven typical glazing systems.	**-ACP:** Melanopic illuminances, relative melanopic efficacy coefficient, circadian stimulus. **-OP:** A man sitting 1.45 m from the window in the middle of the office with a cornea height of 1.2 m.	**-TA:** Measurements and computer-based simulations. **-PA:** The measurements took place on the 28th of February between 10:00 and 14:00 and on March 3rd, 2019 between 10:00 and 11:00 in clear sky conditions. Simulations were performed by means of ALFA, a Rhinoceros plug-in. They were repeated on the 21st of March for clear and overcast sky types between 7:00 and 18:00. For the simulations, three glazing types and three wall colors of equal reflectance were selected among those used in the experimental analysis.
[54]	2020	**-GG:** To verify the feasibility and the potential of a fiber optic daylighting system (FODS) to deliver circadian light in a windowless office in Beijing. **-IAF:** Daylight transporting systems.	A simulated typical office room, dimensions: 6∙3∙3 m; reflectance values of the room surfaces: 0.2 (floor), 0.5 (wall) and 0.7 (ceiling).	**-L:** Beijing. **-WF:** The room was daylit by FODS arranged in three different layouts (10, 24 and 40 devices) to obtain different illuminance distributions at the work plane.	**-ACP:** CS at the observer’s position. **-OP:** Four different positions located in a corner of the office, simulating a sitting man looking alternatively towards the two opposite walls.	**-TA:** Measurements and computer-based simulations. **-PA:** Luminous flux and SPD (Spectral Power Distribution) of light at the end of the FODS were measured by connecting the 9 m optical cables to an integrating sphere. Measurements were done during normal working hours (8:00–17:00) from March 1, 2008 to January 31, 2009 with a 15 min time step. The FODS were simulated in DIALux as luminaires characterized by a compatible photometry provided by the FODS manufacturer and by setting as luminous flux the values measured in the integrating sphere.

**Table 3:** Papers analyzing factors related to technological and interior design focusing on electric light.

Ref.	Year	Goal: (GG) General Goal (IAF) Investigated Architectural Factors Affecting Circadian Response	Methodology			
			Description of the Case Study	Light Source Characteristics: (L) Location (WF) Window Features (LF) Luminaires Features	Evaluation of Circadian Effects: (ACP) Adopted Circadian Parameter (OP) Observer’s Position	Analysis Method: (TA) Type of Analysis (PA) Protocol of Analysis
[70]	2017	**-GG:** Development of Color Quality Assessment Tool (CQAT), based on the luminous flux transfer method, that, considering both the spectral reflectance of interior finishes and the SPD of luminaires, calculates the spectral luminous exitance radiated by interior finishes and the spectral distribution of the light entering the field of view of an observer considering a specific direction and position. **-IAF:** Spectral reflectance of indoor finishes.	Cubic space 3∙3∙3 m wide. The walls were covered in different colors (red and blue) and colors were distributed in the space in different ways (for example, only the frontal wall is colored, and the rest is neutral, or all the walls are colored, etc.).	**-LF:** One LED panel, luminous flux 3600 lm, CCT (Correlated Color Temperature) equal to 6000 K and 2800 K, alternatively.	**-ACP:** CAF (Circadian Action Factor) at the observer’s eye level. **-OP:** A man sitting with his back to a wall looking at the wall in front of him.	**-TA:** Computer-based simulation. **-PA:** Simulations were performed by means of the Color Quality Assessment Tool (CQAT).
[71]	2018	**-GG:** Validation of the software developed in [70] by means of comparison with data measured in a full-scale mock-up. **-IAF:** Spectral reflectance of indoor finishes.	Cubic space 3∙3∙3 m wide. Ceiling and floor were neutral and the perimetral walls colored in gray, red and blue, alternatively.	**-LF:** One LED panel, alternatively set to 6300 K/2420 lm and to 3100 K/2290 lm.	**-ACP:** CAF at the observer’s eye level. **-OP:** A man sitting with his back to a wall looking at the wall in front of him.	**-TA:** Measurements and computer-based simulation. **-PA:** Field spectral measurements were used to validate the Color Quality Assessment Tool (CQAT).
[72]	2017	**-GG:** Analysis of the impact of using different wall colors and light scenes on indoor lighting quality. **-IAF:** Color of interior surfaces.	A neutral laboratory environment (dimensions 2.79∙2.48∙2.70 m) in which a desk was located. The spectral reflectance of the wall located in front of the desk was changed by attaching cardboard of the following colors: white, yellow, light blue, violet, pink, peach and pale blue.	**-LF:** Four LED luminaires (nominal luminous flux 1824 lm) regulated to obtain an average illuminance equal to 300 lx at the desk. The CCT of the luminaires was changed to obtain four light scenes: 2700 K, 3000 K, 4500 K and 6500 K.	**-ACP:** Cyanopic, melanopic, rhodopic, chloropic and erythropic illuminances according to the Irradiance Toolbox [73]. **-OP:** A man sitting at a desk (1.2 m from the floor) looking forwards and 45° left and right. For each one of these directions, measurements were repeated for different tilt angles (0°, 15° and 15° downwards) to simulate different positions of the observer’s head.	**-AT:** Field spectral measurements. **-PA:** Measurements were repeated for each analyzed color and each light scene.
[52]	2018	**-GG:** Proposal of rule-of-thumb equations to calculate the average indirect corneal illuminance (i.e., corneal illuminance due to reflected light) at standing or sitting positions to guide circadian lighting design and their validation through comparison with computer-based simulations. **-IAF:** Total reflectance of indoor finishes.	A room measuring 3.0∙4.8∙3.2 m, simulated according to four different settings: 1) a windowless space where the reflectance was the same for all surfaces. In this case, the following parameters were changed: surface reflectance and the electric light arrangement; 2) a windowless room with different combinations of ceiling, wall and floor reflectance; 3) a room with a window where different values of reflectance for the surfaces, various WWRs and surface reflectance were set (in this case, daylight was not considered); 4) a room with a window where various surface reflectance, WWRs (Window to Wall Ratios) and geographic locations were considered (in this case, only daylight was considered).	**-LF:** Different positions, directions of emission and arrangements of luminaires were considered. **-L:** Helsinki and Shanghai. **-WF:** The window was located on the long side of the room oriented towards south, WWR from 10% to 100% with 0.1 steps were considered.	**-ACP:** Average indirect corneal illuminance; average direct corneal illuminance. **-OP:** A grid at 1.2 m and at 1.6 m from the floor, 0.2 m of spacing between the points and 0.5 m from the perimeter was used. In each point, the illuminance was defined for eight different principal directions, then the average value for each position was defined. Finally, the average for the entire surface was calculated.	**-TA:** Computer-based simulations. **-PA:** Simulations were performed by means of Honeybee and Ladybug in Grasshopper and Daysim for daylight. In case of daylight overcast sky, 22 December at 11 a.m. was considered.

One of the aspects considered in this section is the spectral reflectance of indoor surfaces. When circadian effects are investigated, the evaluation of lighting must be done considering the light reaching the observer’s eyes, going beyond the traditional design approach based on the evaluation of the illuminance at the work plane. The overall illuminance at the eye can be divided into two portions: the direct one, coming directly from the source, and the indirect one determined by the multiple inter-reflections among surfaces. Considering circadian effects of light, due to the role of the spectrum in assessing them, the capability of architectural surfaces in modifying light sources’ SPDs cannot be forgotten. Generally, the attention paid to the aspects of light color is limited to the choice of CCTs [71], forgetting that only if the interior finish color is achromatic, the SPD radiated by the luminaires is similar to the spectrum reaching the observer’s eye. However, if the interior finish color is chromatic, the observer may perceive a luminous environment that is different from the intended design [70]. Of course, the effect of reflectance surfaces applies to daylight as well. Based on these observations, several works reported in Table 2 and Table 3 [52,55,65,66,67,70,72] investigate this aspect, analyzing it both with experimental and numerical research approaches.

Among experimental studies, those performed by a research group from Bratislava [65,66,67] deserve to be cited. In these papers, the authors perform daylight measurements (in some cases by means of a spectroradiometer, in others with a Daysimeter) in scale models representing offices characterized by a rectangular and elongated shape, equipped with a window located on their short side. Measurements are not continuous but collected in specific moments of the day, in overcast sky conditions. The indoor surfaces of the models are characterized by different spectral reflectance values and measurements are conducted at eye level at points located different distances from the window, considering various directions of view, for evaluating non-visual characteristics of light in the entire room. In the cited papers, the authors compare different interior finishes. Both in [65] and in [66], the floor of the models is dark brown, the ceiling white and the perimetral walls were painted in different colors: white (the reference case), yellow, light gray and blue in the former study; white (the reference case), yellow, orange and red in the latter one. In [65], it is demonstrated that all three of the colors determine lower CS values compared to the reference case. The worst results were obtained with yellow, followed by blue and gray. Variations are strongly affected by the observer’s position: the higher the distance from the window, the higher the reduction in the CS values. Moreover, CSs referring to points directed towards the wall are lower than those directed towards the window. In [66], it is found that the use of the different indoor colors determines a reduction in the CS values equal to 6%, 9% and 12% for the red, orange and yellow cases, respectively. An experiment performed in [67] is similar to the one in [65], but in this case, measurements are conducted in winter instead of summer and the reference model has completely white internal surfaces (floor and ceiling included). Again, in this case, all three colors cause a reduction of the CS compared to the reference white case. The worst results are obtained with yellow, followed by gray and blue. The effect of the observer’s position and of the direction of view is confirmed. These works, as do others cited in the previous paragraph, confirm the importance of the correct location in the spaces of workplaces. 

The spectral reflectance of outdoor surfaces facing the windows of the rooms’ object of analysis can have a role in modifying daylight SPD. This topic is treated in the already cited [65], where the experiment already described is repeated by locating the model of another edifice in front of the principle model, obstructing daylight and creating a shading angle of 30°. This model is colored in light brown. General observations done for the non-obstructed case are confirmed, but reductions in CS values are higher. Moreover, in this case, the blue color is characterized by CS values lower than for the yellow. This is probably due to the spectral characteristics of the entering light (the obstruction does not reflect short wavelengths) and, indeed, illuminances at eye level are lower than the other cases. A similar experiment is repeated by the same research group in [55] and, in this case, the obstructing model is colored once in white and once in dark red and continuous daylight measurements were performed from 1st to 25th September 2013. It is found that the ratio of circadian stimulus in the model room shaded by a dark red obstruction to the circadian stimulus in the room shaded by a white obstruction slightly varies day by day because of the different spectral composition and intensity of daylight during each day. 

In [72], the effect of the spectral reflectance of indoor walls is analyzed in a full-scale room, but in this case, electric light is considered. Spectral irradiance measurements were performed at the eye level of a man seated at a desk located in a neutral laboratory environment. The spectral reflectance of the wall located in front of the desk was changed by attaching cardboard of the following colors: white, yellow, light blue, violet, pink, peach and pale blue. The room was lit by four LED luminaires (illuminance at the desk equal to 300 lx) the CCT of which was changed to obtain four light scenes: 2700 K, 3000 K, 4500 K and 6500 K. The impact of light on the circadian system is evaluated by means of the Irradiance Toolbox [73]. It is demonstrated that the E_cyan_, E_mel_ and E_rhod_ values vary depending on the front wall color and on the light scene. In more detail, the greatest differences were found between the base case and violet cardboard, but between pale blue and yellow cardboard and between the light blue and peach one, differences are also significant. Conversely, white and pink cardboard always show almost coincident values.

Other studies investigate the analyzed topic with a numerical approach. In this case, electric light is considered. In [70], the authors develop software called Color Quality Assessment Tool (CQAT), based on the luminous flux transfer method, that, considering both the spectral reflectance of interior finishes and the SPD of luminaires, calculates the spectral luminous exitance radiated by interior finishes and the spectral distribution of the light at the eye level of an observer, considering a specific direction and position. The tool has been validated in a subsequent study by means of comparison with data measured in a full-scale mock-up [71]. In the first study, a cubic space 3∙3∙3 m wide is analyzed. The walls are covered in different colors (red and blue) and colors are distributed in the space in different ways (for example, only the frontal wall is colored, and the rest is neutral, all the walls are colored, etc.). The scene is lit by one LED panel, with CCT equal to 6000 K or 2800 K. In the second study, the analyzed space has the same dimensions, but the ceiling and floor are neutral and the perimetral walls colored in gray, red or blue. The scene is lit by one LED panel, set to 6300 K/2420 lm or to 3100 K/2290 lm. In [70], it is proven that the component of the light incident at the observer’s eye level directly emitted by the luminaire is equal to about 70% for both CCTs and the remaining 30% is due to surface reflections. This demonstrates the importance of accounting for the impact of the environment in modifying spectral reflectance of light emitted by luminaires. From the measurements performed in [71], it is registered that the use of red for the walls causes a reduction of the CAF equal to 1.3% and to 2.5% with a 6300 K LED, whereas the use of blue for the walls causes an increase in the CAF equal to 8.4% and to 6.9% with a 6300 K LED. 

The importance of defining the effect of indoor surface reflectance is confirmed in [63] as well. The goal of the paper is to propose rule-of-thumb equations to calculate the average indirect corneal illuminance (i.e., corneal illuminance due to reflected light) at standing or sitting positions to guide circadian lighting design. The equations are validated through comparison with computer-based simulations performed with Honeybee and Ladybug in Grasshopper and Daysim for daylight. 

Another topic analyzed by researchers is the effect of the spectral transmittance of glazing systems in modifying the SPD of daylight entering buildings. 

In [68], continuous spectral measurements are performed during the day at the observer’s eye level in a full-scale office room located in Beijing characterized by neutral surfaces and equipped with a window facing south, and equipped alternatively with seven types of glazing: clear, blue, bronze, gray, green, dark blue and red. The experiment demonstrates that the dark blue glazing causes very low CS values (< 0.3) across all times in comparison to other glazing types, and the CS values of other glazing types follow a similar variation: they start to rise at 08:30 and achieve a plateau from 10:45 to 14:30, and then go down towards late afternoon (16:00). 

In [69], the effect of the transmittance of glazing is analyzed together with the spectral reflectance of the interior wall and the different solutions are evaluated by means of ALFA software. The case study is a typical cellular office in Ljubljana. The indoor wall colors are alternatively purple, blue, green, red, orange and gray at three different reflectance levels. The room is daylit by a single north-oriented window alternatively equipped with seven typical glazing systems. It is found that low-e glazing with high visual transmittance and blue walls are the combinations with the highest non-visual entrainment, while the opposite is true for the combination of bronze-tinted solar protective glazing and orange walls. In general, a better non-visual environment can be achieved using materials characterized by higher spectrally neutral transmissivity or reflectance compared to those characterized by spectrally non-neutral properties and lower transmissivity or reflectance.

Another study worth citing is [54] since it focuses on a poorly studied topic. Its goal is to verify the feasibility and the potential of a fiber optic daylighting system (FODS) to deliver circadian light in a windowless office in Beijing. The case study is a simulated typical office room, daylit by FODS arranged in three different layouts (10, 24 and 40 devices) to obtain different illuminance distributions at the work plane. The FODS are simulated in DIALux as luminaires characterized by a compatible photometry provided by the FODS manufacturer and by setting as luminous flux values those measured by means of an integrating sphere for different daylight conditions. Spectral measurements are conducted to obtain the SPD of daylight transported by the FODS to evaluate the CS. It is proven that the percentage of observed CS values is higher than 0.3 for less than 20% with layout 1, 40% with layout 2 and achieves around 60% with layout 3. The maximum values of CS are found to be 0.42, 0.52 and 0.6 for layouts 1–3, respectively. Differences in circadian light performance between various positions tend to become lower as the horizontal illuminance increases.

## 5. Conclusions

The paper has underlined the fundamental role of architectural design in defining the quantity and the quality of light received by people during the day. However, it has highlighted how complex it is to evaluate the mutual interactions between light and architecture to define their effect on circadian systems. Indeed, despite the efforts of researchers in analyzing the different themes of this review, it appears clear that comparisons among the results of the available studies are difficult and that it is not possible to quantify the weight of each architectural factor in modifying the characteristics of light and the consequent circadian effect. This difficulty is due to the differences in the used methodological approaches and the lack of clear and shared evaluation techniques able to univocally define the influence of light on circadian system regulation.

The most significant problems in this sense turn out to be the following:

1. *Coexistence of multiple metrics to define the circadian effect of light.* As is shown in Table 1, Table 2 and Table 3 and in Appendix A, there are several metrics adopted by researchers and they are not comparable to one another.
2. *Absence of limit thresholds for the evaluation parameters.* For each of the mentioned metrics, it is difficult to define the corresponding limit thresholds that are essential to understand if a light stimulus is beneficial or not to regulating the circadian system. Moreover, considering that one of the key characteristics of circadian lighting is the timing, these thresholds should be different depending on the time of day. An attempt in this sense has been made recently for the CS and for the equivalent melanopic illuminance. According to [74], a CS value of 0.3 or higher was recognized to reduce sleepiness and increase alertness in workers, so it would be desirable to achieve this value in the morning. The WELL standard [75] proposes that an equivalent melanopic illuminance equal to or higher than 200 should be achieved between 9:00 and 13:00 for a minimum of 75% of workstations in an office. However, these thresholds should be further verified.
3. *Necessity of a spectrally based analysis approach.* Irrespective of the parameter adopted to evaluate the circadian effect, an assessment of non-visual conditions presumes a spectrally based analysis, whereas the common design practice is limited to a visual and photopic approach, often focused exclusively on the evaluation of illuminance patterns. This brings the two further issues.
4. *Scarce spread of calculation software able to evaluate light accounting for its spectral characteristics.* As it can be seen in Table 1, Table 2 and Table 3, software for a spectrally based evaluation of light has started to appear on the scene. They simulate the SPD of light sources and the inter-reflections between light and surfaces, accounting for their spectral properties. For example, as mentioned in the previous paragraph, Kim et al. [70] have proposed an evaluation tool for indoor luminous environments, including circadian light analysis for electric light. In 2015, Inanici et al. [50] released a Grasshopper plug-in called LARK [76], useful for evaluating circadian effects of light by performing nine-channel simulations, allowing the spectral distribution of the sky to be modeled. In 2018, Solemna released a more sophisticated multi-channel simulator, Adaptive Lighting for Alertness (ALFA) a radiance-based plug-in for Rhinoceros able to perform 81-channel simulations [77], allowing the spectral simulation of both daylight and electric light. These calculation tools are not yet widespread and are mainly used for research purposes. Moreover, due to the complexity of a spectrally based evaluation, simulations are performed only with a static calculation approach.
5. *Difficulty in collecting spectra of daylight sources.* Even using software like those described in point 4, it is necessary to know the spectral reflectance values of the surfaces and the SPDs of the sources that are input data for the simulations. As for the materials, generally, software provides sufficiently substantial archives containing spectral reflectance data and, regarding the electric light, obtaining SPDs from manufacturers is not really difficult. The urgent problem is the modeling of daylight SPD. It continuously changes depending on time due to weather conditions, time of day, season and location [51,78,79]. Available calculation tools apply a simplified modeling approach: LARK simulates the spectral distribution of the sky starting with measured spectral irradiance data (sample measurements can be downloaded from the LARK website itself), whereas the sun is modeled as a non-spectral, equal energy white source. As an alternative to measured data, it is possible to use as input the sky CCT, from which the corresponding CIE D standard illuminant is identified. So, for an accurate simulation, the user should know the CCT trends of daylight during the specific day of the design location. ALFA calculates the sky and the sun spectral characteristics depending on the selected location, according to the US Air Force Geophysics Laboratory’s (AFGL’s) standard mid-latitude summer profile [80]. Four different sky typologies are available: clear, overcast, hazy, heavy rain clouds. The limitation of the software is the use of a generic spectral sky model [81]. Moreover, as previously mentioned, such software does not allow the performance of dynamic analyses. To obtain a dynamic evaluation of daylight availability referring to the entire year, as can be read in the tables, researchers have proposed different approaches, all based on the same concept [16,27,49,62,63]; knowing the daylight SPD, it is possible to calculate the photopic illuminances corresponding to specific levels of circadian stimulation for the analyzed SPD. Consequently, taking the D illuminants as daylight SPDs, the entire evaluation can be performed in terms of photopic illuminances. This approach has two limitations: it highly simplifies the modeling of daylight SPD (only two or three D illuminants [16,27,49] or an average spectrum based on typical weather conditions [62,63] are used); the interaction among surfaces is not considered from a spectral point of view, since it is assumed that the spectrum of light reaching the eye coincides with that of the D illuminant. In some cases, a simplified approach is proposed, like in [63], where the spectrum at the eye level of students is the product of the spectrum of the source for the spectral reflectance of the desk. However, this approach is also limited, since it considers only the interaction between the surfaces supposed to occupy most of the visual field.
6. *Difficulty in performing measurements.* For existing buildings, the evaluation of effect of architectural parameters on circadian stimulation could be performed by means of field measurements. However, the instruments are not cheap and, especially in the presence of daylight, the repetition of the measurements during the day to detect natural variations can be onerous.

As the open questions about the proper evaluation of the circadian impact of light are so numerous, a precise assessment of the role of architecture in defining the non-visual effects is a complex task. However, from the reviewed works, it can be stressed that the solutions suggested to maximize non-visual comfort are mostly similar to those to maximize visual comfort: correct position of the building, optimization of the window-to-wall ratio depending on the location, maximization of daylight entrance that must be considered the prior light source and the use of highly reflective and neutral materials to increase the indoor light levels and balance light distributions. This confirms the fact that the different effects of light on people’s wellbeing are strictly connected, reaffirming the necessity of the spread of an integrative light culture, considering all the aspects of lighting in a synergic way, with the goal to design spaces to live well, and consequently to sleep well.

## Data Availability

Not applicable.

## Appendix A

**Table A1:** Definitions of circadian performance parameters.

Parameter	Acronym	Definition
Circadian Daylight Autonomy	DA_circadian_	Daylight autonomy is the percentage of the year during which a target illuminance is achieved by daylight alone [82]. Generally, the set target illuminance coincides with the average daylight illuminance corresponding to the specific visual tasks according to the standards [83]. However, to evaluate circadian effects, the target can correspond to a specific level of stimulation of the circadian system. Cajochen et al. [12] found that exposure to about 300 lux to 4100 K polychromatic fluorescent light at night was required to maintain maximal alertness. Through a conversion method, in [27], the circadian equivalent illuminance threshold for D illuminants were calculated: 190 lx for the D65 and 180 for the D75. Then the DA_circadian_ was calculated at eye level, referring to these thresholds.
Non-Visual Effect	N_VE_	Based on the study by Cajochen et al. [12] and Phipps-Nelson [13], in [16], a ramp function reporting the non-visual effect, N_VE_, of daylight as a function of the visual illuminance (considering a D55 illuminant) is described. This N_VE_ is 0 when the illuminance is lower than 210 lx, 100% when the illuminance is higher than 960 lx and evaluable by means of a linear function when the illuminance is between these two thresholds.
Cumulative Annual Non-Visual effect	C_NVE_	Starting from the N_VE_, it is possible to evaluate the cumulative annual non-visual effect, C_NVE_, for a specific series of data (for example, the results of a dynamic daylight simulation). It is the average of the N_VE_ of the series itself [16].
Circadian Action Factor	CAF	The CAF is the ratio of circadian and visual stimulus calculated as: $CAF=\frac{\int_{380}^{760}C\left(\lambda \right)P\left(\lambda \right)\delta \lambda }{\int_{380}^{760}V\left(\lambda \right)P\left(\lambda \right)\delta \lambda }$ where C(λ) is the circadian spectral sensitivity function, V(λ) is the photopic visual sensitivity and P(λ) is the SPD of a light source [84].
Circadian Light	CL_A_	Circadian light is the irradiance at the cornea weighted to reflect the spectral sensitivity of the human circadian system as measured by acute melatonin suppression after a 1 h exposure [85].
Circadian Stimulus	CS	Circadian stimulus is the calculated effectiveness of the spectrally weighted irradiance at the cornea from threshold (CS = 0.1) to saturation (CS = 0.7), assuming a fixed duration of exposure of 1 h [85].
Circadian Stimulus Autonomy	CSA	The percentage of time throughout the year when a specific threshold of circadian stimulus is met by daylight [62]. The calculation procedure of CSA is based on daylight autonomy, since by knowing the spectral power distribution (SPD) of the perceived light (generally assumed to be coincident with a D illuminant), the illuminance threshold corresponding to a specific desired CS value can be defined. Usually, CSA is calculated considering a CS value of 0.3, according to the recommendation of [74].
Cyanopic, Melanopic, Rhodopic, Chloropic and Erythropic Illuminances.	E_cyan_, E_mel_, E_rhod_, E_chlor_, E_eryth_.	Equivalent illuminances for the different photoreceptors in the human eye: cyanopic illuminance, which relates to cyanolabe and therefore to short wavelength-sensitive (S) cones; melanopic illuminance, which relates to melanopsin and therefore to ipRGCs; rhodopic illuminance, which relates to rhodopsin and therefore to rods; chloropic illuminance, which relates to chlorolabe and therefore to medium wavelength-sensitive (M) cones; erythropic illuminance, which relates to erythrolabe and therefore to long wavelength-sensitive (L) cones. They are evaluated by means of the Irradiance Toolbox [73].
Relative Melanopic Efficacy Coefficient	RME	The ratio of equivalent melanopic illuminance to photopic illuminance [69].
